# Crx Is Posttranscriptionally Regulated by Light Stimulation in Postnatal Rat Retina

**DOI:** 10.3389/fcell.2020.00174

**Published:** 2020-04-07

**Authors:** Yihui Wu, Jin Qiu, Shuilian Chen, Xi Chen, Jing Zhang, Jiejie Zhuang, Sian Liu, Meng Yang, Pan Zhou, Haoting Chen, Jian Ge, Keming Yu, Jing Zhuang

**Affiliations:** State Key Laboratory of Ophthalmology, Zhongshan Ophthalmic Center, Sun Yat-sen University, Guangzhou, China

**Keywords:** endogenous Crx expression, posttranscriptionally regulation, cone rod homeobox (Crx), retinal development, light simulation

## Abstract

Cone rod homeobox (Crx) plays a key role at the center of a regulatory network that coordinates many pathways in the retina. Its abnormal expression induces retinal disorders. However, the underlying regulatory mechanism of Crx expression is not well defined. Here, we present data that show that the levels of Crx mRNA were inconsistent with that of Crx protein in primary retinal neurocytes cultured in light conditions. Crx protein levels were significantly higher (2.56-fold) in cells cultured in the dark than in cells cultured in light, whereas Crx mRNA was not changed in either type of cell. Moreover, the expression of Crx protein showed a significant light intensity-dependent decrease. Consistently, Crx downstream genes rhodopsin and arrestin also decreased in retinal neurocytes upon light exposure. Furthermore, Crx promoter activity assay performed in primary retinal neurocytes further indicated that light exposure and darkness did not affect its inducibility. In addition, the inconsistency between Crx mRNA and protein expression after light exposure was not observed in 661w cells transfected with plasmid pcDNA3.1-Crx, suggesting that the inconsistency between Crx mRNA and protein induced by light was specific to the endogenous Crx. More importantly, this observation was confirmed *in vivo* in postnatal day 15 (P15) retinas but not in adult retinas, further implicating that the posttranscriptional regulation mechanism may be involved in Crx expression in the developing retina. Therefore, our study sheds light on the mechanism of Crx expression in postnatal rat retina.

## Introduction

The homeodomain transcription factor Crx encodes a 299-amino acid residue protein with a specific paired-like homeodomain at its N terminus (Chen et al., 1997). Crx is exclusively expressed in retinal photoreceptors, RPE cells, and pinealocytes (Yanagi et al., 2002; Lee et al., 2010; Read et al., 2014). It binds to the sequence TAATCC/A and transactivates downstream photoreceptor-specific genes, including rhodopsin, cone opsin, and arrestin, in many species (Furukawa et al., 1997). Substantial evidence indicates that Crx plays an important role in the regulation of photoreceptor cell differentiation and development. A study by Mansergh et al. (2015) demonstrated that 365 genes are upregulated and 385 genes are downregulated by Crx in the retina. Crx knockout mice exhibit a thinner outer segment and severely abnormal synaptic endings in the outer plexiform layer (Morrow et al., 2005). Crx mutations may lead to many diseases, such as retinitis pigmentosa (RP), cone-rod dystrophy (CORD), and Leber congenital amaurosis (LCA) (Freund et al., 1997; Swain et al., 1997; Hull et al., 2014).

Additionally, the development of the visual system is a complex process that involves multisystem cooperation at different time points. Both genomic abnormalities and many environmental elements may interrupt this organized process (Brémond-Gignac et al., 2011; Ackman et al., 2012). During a certain period after birth, external factors, such as proper light stimulation, affect normal development. Crx expression levels vary at different developmental stages (Rath et al., 2007; Read et al., 2014). Abnormal Crx expression may induce retinal diseases. For example, the overexpression of retroviral Crx in developing retinal cells results in a marked increase in rod photoreceptors and an almost complete absence of amacrine interneurons. The differentiation of rod outer segments and terminals is dramatically impaired (Mansergh et al., 2015). Another study also demonstrated that the severity of retinitis pigmentosa in mouse models was associated with the overexpression of opsin (Tan et al., 2001). Therefore, these lines of evidence highlight the importance of this transcription factor in morphogenesis and the determination of cell function. Abnormal Crx expression might set off a cascade of abnormal reactions and ultimately cause retinal dysfunction. Thus, the elucidation of its expression mechanism in the retina is a prerequisite for the development of retinal disorder therapies (Soto et al., 2012).

In the pineal gland, Crx mRNA exhibits a daily expression rhythm (Sakamoto et al., 1999). The rhythmic nature of pineal Crx protein expression may directly modulate the daily concentration profile of AANAT by inducing the nighttime expression of this enzyme, thus facilitating nocturnal melatonin synthesis and playing a role in ensuring the correct tissue distribution of AANAT (Rohde et al., 2014). However, as yet, there have been few studies on the expression profile of Crx in the retina, and the results of these studies have been inconsistent. For example, a study by Katsuhiko indicated that Crx mRNA levels in the adult rat retina are constant under a daily light-dark cycle. However, the expression of the downstream NAT genes exhibits a circadian rhythm. Thus, they suggested that Crx expression in the retina is regulated by posttranscriptional regulatory mechanisms (Sakamoto et al., 1999). Another study revealed that Crx increases over time during zebrafish retinal development [24–72 h post-fertilization (hpf)]. After 72 hpf, however, a significant decrease in Crx mRNA expression is observed, followed by the emergence of a circadian rhythm, and this rhythm is maintained in adults (Laranjeiro and Whitmore, 2014). However, all studies on Crx have focused on the circadian rhythm of the mRNA levels, but not protein levels, of Crx in the retina. Thus, the exact regulatory mechanism of Crx expression in response to light stimulation in the retina is poorly understood.

To answer this question, we elucidated the connection between Crx and light exposure both *in vivo* and *in vitro* to provide new insight into the mechanism of Crx expression in the retina.

## Materials and Methods

### Cell Culture

Primary rat retinal neurocytes were cultured as described previously (Zhuang et al., 2008). Briefly, 1-day-old SD rats (provided by the animal center of the Zhongshan Ophthalmic Center, Sun Yat-sen University, Guangzhou, China) were sacrificed by intraperitoneal injection of Nembutal (60 mg/kg, Sigma, St. Louis, MO, United States). The retinas were first separated from the enucleated eyeballs and then incubated in a solution containing 0.125% trypsin for 20 min at 37°C to obtain a suspension of single cells. The cells were seeded at a density of approximately 1 × 10^6^ cells/ml on a culture plate precoated with 0.01% poly-L-lysine for 2 h and incubated in a humidified atmosphere of 5% CO_2_ and 95% air. Twelve hours later, the cells were treated with 10 μm/ml Ara-C (Sigma, St Louis, MO, United States) to suppress the growth of non-neurocytes. After 12 h, the medium was substituted with neurobasal medium (Carlsbad, CA, United States). The cultured cells were characterized by double-staining with anti-Map2 (Boster, Wuhan, China) and anti-Crx antibodies (Abcam, Cambridge, United Kingdom).

Three days after culture, the cells were divided into five groups: group 1, which included four dishes of primary retinal neurocytes obtained from the same rats and incubated in darkness (D/D) or a cycle of 12 h of daylight and 12 h of darkness (L/D) for 24 h, group 2, which included four dishes of primary retinal neurocytes obtained from the same rats and incubated in darkness (D/D) or a cycle of 12 h of daylight and 12 h of darkness (L/D) for 48 h, group 3, which included four dishes of primary retinal neurocytes obtained from the same rats and incubated in darkness or daylight for 24 h, group 4, which included two dishes of primary retinal neurocytes obtained from the same rats and incubated in light for 12 and 24 h, respectively, and group 5, which included eight dishes of primary retinal neurocytes obtained from the same rats and incubated in darkness or 200 luc, 400 luc, 600 luc, or 800 luc of daylight for 24 h. At different time points, mRNA and protein were collected for analysis.

Mouse retinal 661W cells were purchased from ATCC (Manassas, VA, United States) and cultured in Dulbecco’s modified Eagle’s medium (DMEM, Gibco, CA, United States) supplemented with 10% fetal bovine serum (FBS; Gibco, CA, United States) and 1% penicillin/streptomycin (Gibco, CA, United States) in a humidified 5% CO_2_ incubator.

All cells were cultured in 600 luc of daylight unless otherwise indicated.

### Real-Time RT-PCR

Total RNA was isolated with TRIzol reagent (Invitrogen, Carlsbad, CA, United States). One microgram of total RNA was subjected to reverse transcription with the PrimeScriptTM RT-PCR kit (Takara, Dalian, China) according to the manufacturer’s protocol. Real-time PCR was employed to measure Crx, rhodopsin, and arrestin expression using the SYBR Green system (Roche, Indianapolis, IN, United States). The following primer pairs were used: rat Crx, 5′-ATGCACCAGGCTGTCCCATAC-3′ and 5′-CACATCCGGGTACTGGGTCTT-3′; rhodopsin, 5′-GCAGTGTTCATGTGGGATTG-3′ and 5′-CTGCCTTCTGA GTGGTAGCC-3′; arrestin, 5′-GTGTCATACCATATCAAAGG TAAGC-3′ and 5′-GGAACGGCACCTCAGTAGC-3′; exogenous Crx, 5′-TGTCAGGCCAGAGCTATAGC-3′ and 5′-AGATCCTC TTCTGAGATGAG-3′; mouse Crx, 5′-GGACCTGATGCAC CAGGCTG-3′ and 5′-GTTTCTGCTG CTGTCGCTGC-3′; rat β-actin, 5′-TCACCCACACTGTGCCCAT-3′ and 5′-TCTT TAATGTCACGCACGATT-3′. Mouse β-actin (5′-A GGT CATCACTATTGGCAACG-3′ and 5′-ACGGATGTCAACGTC ACACTT-3′) was used as an internal control. The mRNA expression of the target gene relative to that of the internal control gene, β-actin, was calculated using the ΔCT method as follows: relative expression = 2^–Δ^ ^*CT*^, ΔCT = CT (target gene)-CT (β-actin). The data were analyzed in triplicate.

### Western Blot Assay

Western blotting was performed by following standard protocols. Cells and retinal tissue were lysed with RIPA buffer supplemented with PMSF. Whole protein was separated on a sodium dodecyl sulfate/polyacrylamide electrophoresis gel and transferred to a nitrocellulose polyvinylidene fluoride membrane. The following primary antibodies were used: rabbit anti-Crx (1:1000, Abcam, Cambridge, United Kingdom), rabbit anti-β-tubulin (1:1000, Santa Cruz Biotechnology, Inc., Dallas, TX, United States), rabbit anti-GAPDH (Proteintech, Chicago, IL, United States; diluted 1:20000), mouse anti-rhodopsin (1:1000, Abcam, Cambridge, United Kingdom), and mouse anti-arrestin (1:1000, Abcam, Cambridge, United Kingdom). The proteins were visualized with horseradish peroxidase (HRP)-conjugated anti-rabbit and anti-mouse IgG (Proteintech, Chicago, IL, United States), and the protein bands were then detected using an enhanced chemiluminescence detection system.

### Immunohistofluorescence Analysis of Cells

Immunohistofluorescence assay was carried out as per standard protocol. The primary retinal neurocytes cultured in light and darkness for 24 h were fixed with 4% paraformaldehyde for 15 min, permeabilized with 0.1% Triton X-100 (Sigma, St Louis, MO, United States) for 10 min, and blocked with 10% normal goat serum for 30 min. Then, the neurons were double stained with rabbit anti-Crx (1:100, Abcam, Cambridge, United Kingdom) and mouse anti-Map2 (1:100, Boster, Wuhan, China); rabbit anti-H2AX (1:100, Cell Signaling Technology, Danvers, MA, United States) and mouse anti-Map2 (1:100, Boster, Wuhan, China); rabbit anti-Arrestin (1:100, Abcam, Cambridge, United Kingdom) and mouse anti-Map2 (1:100, Boster, Wuhan, China); rabbit anti-Rhodopsin (1:100, Abcam, Cambridge, United Kingdom) and mouse anti-Map2 (1:100, Boster, Wuhan, China). DAPI was used to stain nuclei. The percentages of Crx- and Map2-positive cells in DAPI-positive cells were determined in samples from at least three independent experiments. Images from 10 randomly selected fields were used for analysis in ImageJ software. Neurite length was stained with anti-Map2 antibody and quantified using Simple Neurite Tracer in ImageJ software. The neurite length was confirmed in samples from at least three independent experiments. Images from 100 randomly selected Crx-positive neuron cells in the two groups were used for neurite length quantification. For the assay of DNA damage, cells were analyzed for γ-H2AX foci formation by immunofluorescence. The amount of γ-H2AX foci was scored in images obtained using a constant exposure time. At least 400 cells were counted.

### Reporter and Plasmid Construction

A fragment spanning from −2258 to +122 bp relative to the transcription start site of the rat Crx genomic sequence was produced by PCR with the following primers: forward primer 5′-GGGGTACCCCTTGCGCCGCGGCTGGCGCA GC-3′; common reverse primer 5′-GAAGATCTTCAACCTGT AAATCCCAGTCCAG-3′. This fragment was fused to the promoterless firefly luciferase gene of the pGL3-Basic vector (Promega, Madison, WI, United States) to generate a Crx (−2258/+122)-luc. Mouse Crx cDNA was inserted into the pcDNA3.1-Myc vector at the *Kpn*I and *Xho*I enzyme sites to produce an expression plasmid, pcDNA3.1-mCrx-Myc.

### Crx Promoter-Reporter Assay

Primary rat retinal neurocytes were transfected using Lipofectamine 2000 (Invitrogen, Carlsbad, CA, United States) as described previously (Yang et al., 2016). Two micrograms of various reporter plasmids, 2 μg of expression plasmids pcDNA3-based vectors, and 100 ng of a Renilla luciferase reporter plasmid, pCMV-RL (Promega, Madison, WI, United States), were transfected into the cells. The pCMV-RL plasmid encoding Renilla luciferase was included in all the samples to monitor the transfection efficiency. Twenty-four hours post-transfection, the levels of firefly and Renilla luciferase activity were measured sequentially from a single sample using the Dual-Glo Luciferase Assay System (Promega, Madison, WI, United States). The firefly luciferase activity levels were normalized to the Renilla luciferase activity levels.

### MTT Assay

Cell viability was determined by MTT assay 24 h after exposure to daylight or darkness. Cell viability was calculated as the optical density ratio of a treated culture over that of an untreated control.

### *In vivo* Experiment

A total of 18 two-month-old SD rats and 22 postnatal 15-day-old SD rats were obtained from the Ophthalmic Animal Laboratory, Zhongshan Ophthalmic Center, Sun Yat-sen University. All experiments were approved by the Institutional Animal Care and Use Committee of the Zhongshan Ophthalmic Center [Permit Number: SYXK (YUE) 2010-0058]. All animals were reared in large cages at an ambient temperature of 16–26°C and a relative humidity of 40–70%. The rats were dark-adapted from 6:00 pm to 8:00 am before the experiment, and their right eyes were covered with eyepatches.

Then, the rats were treated as shown in Figure 6A. Adult and postnatal 15 (P15) rats were reared in light with one eye covered by an eyepatch. At 4:00pm and 8:00pm, the eyeballs of rats (including 6 P15 and 6 adult rats in each time point, respectively) were enucleated, and the retinal tissues were divided into two parts. One was prepared for RNA assay and the other for Western blot assay. The mRNA and protein level of Crx were compared between the patched and un-patched eyes from the same rat, and the un-patched eyes were used as control. For immunohistofluorescence assay, at 8:00pm, the eyeballs of rats (including 4 P15 and 4 adult rats, respectively) were enucleated, processed to produce ordinary OCT-embedded sections, and sequentially sectioned to produce meridian sections (8 μm thick).

### Immunohistofluorescence Assay

Retinal slides were fixed with 4% paraformaldehyde at room temperature for 20 min and subsequently incubated with 0.5% Triton X-100 for 10 min. The samples were then treated with a blocking solution (10% normal goat serum) for 30 min. The tissue was then stained with rabbit anti-Crx (1:100, Abcam, Cambridge, United Kingdom), while the nuclei were detected with DAPI. The images obtained using a constant exposure time, and the un-patched retinal tissue from the same rat was used as control.

### Statistical Analysis

The data are expressed as mean ± SD. The differences between the mean values were evaluated with a two-tailed Student’s *t*-test (for 2 groups) and analysis of variance (ANOVA; for >2 groups). All calculations and statistical tests were performed by the computer programs Microsoft Excel 2003 (Microsoft, Redmond, WA, United States) or SPSS 11.5 (SPSS, Chicago, IL, United States). *P* < 0.05 was considered significant for all analyses.

## Results

### The Level of Crx Protein Is Consistent With That of mRNA in Retinal Neurocytes Cultured in a Light-Dark Cycle (L/D) or Constant Darkness (D/D)

**FIGURE 1:**
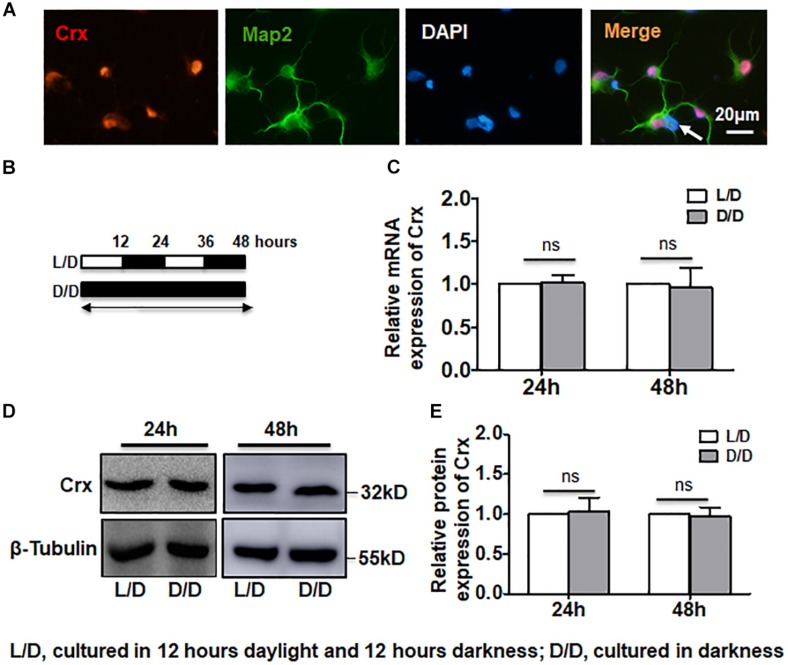
Stable Crx expression in retinal neurocytes cultured in the dark. **(A)** Rat primary retinal cells were double-stained for Crx (red) and Map2 (green). A few Map2-positive cells did not express Crx (white arrow). **(B)** The treatment strategy for analyzing the levels of Crx mRNA and protein in primary retinal neurocytes. Primary cells were cultured on a light-dark (L/D) cycle or in total darkness. **(C)** Crx expression was measured by real-time RT-PCR. There was no difference in Crx mRNA expression between the L/D cycle or total darkness groups 24 or 48 h after treatment (*P* > 0.1). **(D)** After 24 or 48 h of exposure to a light-dark cycle or total darkness, Crx protein was measured by Western blot. **(E)** Relative Crx protein expression in primary retinal neurocytes was quantified by densitometry. The Crx protein levels in primary retinal neurocytes cultured in the dark were the same as that in primary retinal neurocytes cultured on a L/D cycle (*P* > 0.1). All results were confirmed by three independent experiments. The error bars represent the SD of the mean (*n* ≥ 3).

Since Crx is a key regulator of photoreceptor cell differentiation and development (Furukawa et al., 1997), we first analyzed Crx expression in primary retinal cells cultured under a light-dark (L/D) cycle or in constant darkness (D/D). One day after the birth of the rats, primary retinal neurocytes were double-stained for Map2 to verify the identity of these cells. As shown in Figure 1A, 98% of the cells were Map2-positive. Crx and Map2 were coexpressed in most nuclei (Figure 1A). Only a few cells did not express Crx (white arrow). This is consistent with the results of a previous study in which rod photoreceptors comprised over 70% of retinal cells in the postnatal rat retina (Cepko et al., 1996). Then, we divided the cultured cells into two groups. For the constant darkness (D/D) group, the cell culture plates were wrapped with tinfoil (a crack was left between tinfoil and plate for gaseous exchange) to protect the cells from light stimulation, while the light-dark cycle (L/D, 600 lux) group was exposed to 12 h of light and 12 h of darkness (Figure 1B). Both RNA and proteins were extracted and detected at different time points. Quantitative RT-PCR showed that there was no difference between Crx mRNA expression in retinal neurocytes cultured in D/D and in L/D 24 and 48 h after treatment (24 h: L/D, 1; D/D, 1.018 ± 0.084; 48 h: L/D, 1; D/D, 0.959 ± 0.232; *p* > 0.1) (Figure 1C). Moreover, a Western blot assay revealed that the level of Crx protein expression in retinal neurocytes cultured in D/D was also not changed compared with that of retinal neurocytes cultured in L/D. We calculated the expression levels based on the integrated densities and areas of the protein bands. The average ratio of Crx expression to β-tubulin expression in the D/D group was defined as 1.0. The protein level of Crx in retinal neurocytes cultured in D/D was not altered compared with that of retinal neurocytes cultured in L/D after 24 and 48 h (24 h: L/D, 1; D/D, 0.988 ± 0.225; 48 h: L/D, 1; D/D, 1.085 ± 0.148; *P* > 0.1) (Figure 1E). These results suggest that 12 h of daylight stimulation does not affect the Crx mRNA and protein expression levels in retinal neurocytes cultured in D/D and L/D.

### Light Stimulation Induces Inconsistent mRNA and Protein Levels of Crx in Retinal Neurocytes

**FIGURE 2:**
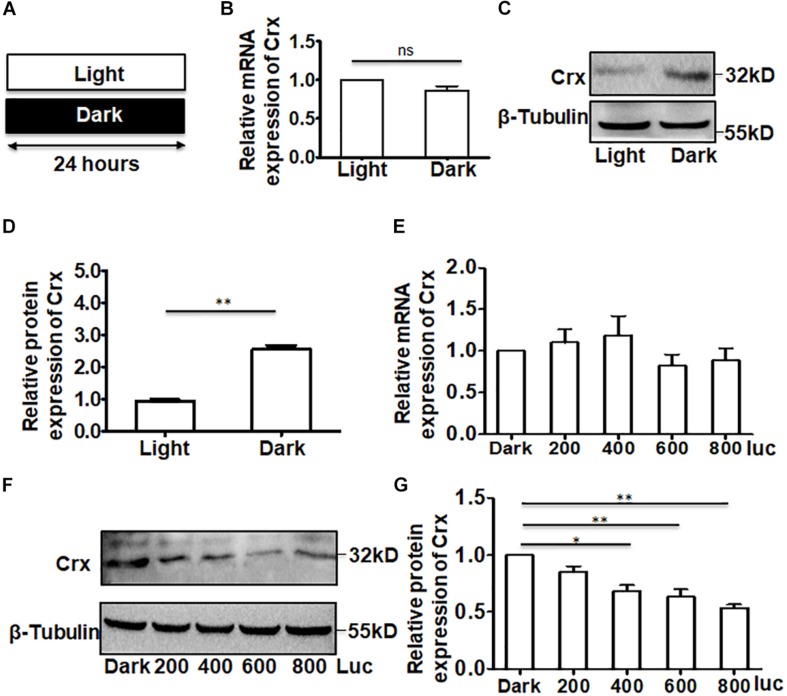
Crx is posttranscriptionally regulated in retinal neurocytes. **(A)** Primary retinal neurocytes were cultured in light (600 lux) or darkness for 24 h. **(B)** The expression level of Crx mRNA in neurocytes cultured in light and darkness was the same, as determined by real-time RT-PCR (*P* > 0.05). **(C)** The upregulation of Crx protein in neurocytes cultured in darkness was compared to Crx protein expression in the controls (neurocytes culture in daylight), as determined by Western blot. **(D)** Crx protein expression in primary retinal neurocytes cultured in darkness was significantly higher than that in the controls (primary retinal neurocytes cultured in daylight) (**P < 0.001). **(E)** Light intensity did not affect the expression of Crx mRNA (*P* > 0.05). **(F)** Increasing light intensity increased the expression of Crx protein. **(G)** A light intensity-dependent decrease in Crx expression in retinal neurocytes is presented in a histogram (*P < 0.05, **P < 0.01). All results were confirmed by three independent experiments. The error bars represent the SD of the mean (*n* ≥ 3). The asterisks represent statistically significant differences between the controls and experimental groups.

We next asked whether the results described above were affected by the time point at which total RNA and protein were extracted from the cells cultured in darkness. To answer this question, we analyzed the Crx mRNA and protein levels in retinal neurocytes cultured in light or total darkness. The primary cells were cultured as shown in Figure 2A. After culturing for 24 h, whole protein and RNA were extracted. Quantitative RT-PCR indicated that light did not affect Crx mRNA levels in retinal neurocytes (light, 1; dark, 0.863 ± 0.101; *P* > 0.05) (Figure 2B). However, the protein expression of Crx was notably increased in neurocytes cultured in darkness compared with in the control (neurocytes cultured in light) by 2.85-fold (light, 1; dark, 2.85 ± 0.45; ^∗∗^*P* < 0.01; Figure 2D). Therefore, these data indicate that the translation or stability of Crx in retinal neurocytes might be affected by light stimulation. To confirm this hypothesis, we cultured retinal cells at different light intensities. Twenty-four hours after treatment, as shown in Figure 2E, there was no difference in the level of Crx mRNA among neurocytes cultured at different light intensities (dark, 1; 200 lux, 1.102 ± 0.273; 400 lux, 1.189 ± 0.398; 600 lux, 0.822 ± 0.233; 800 lux, 0.885 ± 0.254; *P* > 0.1) (Figure 2E). However, a light intensity- and time- dependent decrease Crx protein expression was observed in retinal neurocytes. The decrease in Crx protein level is presented in histograms (dark, 1; 200 lux, 0.742 ± 0.231; 400 lux, 0.648 ± 0.104; 600 lux, 0.574 ± 0.0.152; 800 lux, 0.538 ± 0.0.042; ^∗∗^*p* < 0.01) (Figures 2F,G and Supplementary Figure S1).

### The Expression Levels of Downstream Genes Rhodopsin and Arrestin Are Consistent With That of Crx Induced by Light in Primary Retinal Neurocytes

**FIGURE 3:**
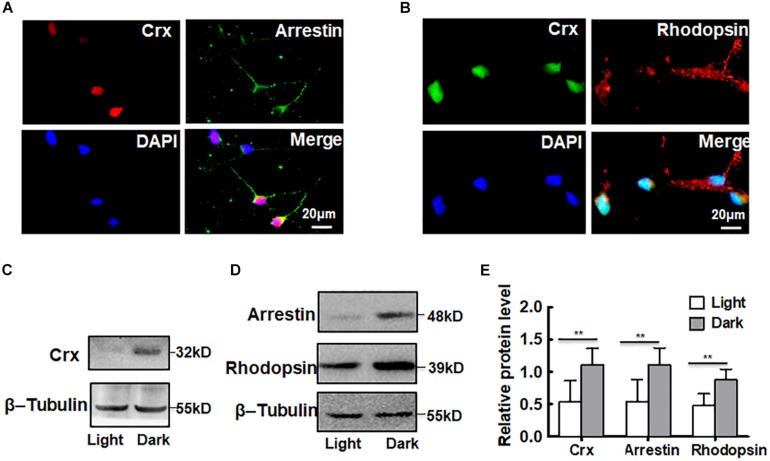
Downstream genes of Crx are also affected by light stimulation. **(A)** Double-staining for Crx (red) and arrestin (green) in retinal neurocytes. Scale bars: 20 μm. **(B)** Double-staining for Crx (green) and rhodopsin (red) in retinal neurocytes. Scale bars: 20 μm. **(C)** Western blotting showed that the protein expression of Crx in primary retinal neurocytes cultured in darkness was remarkably higher than that in controls (primary retinal neurocytes cultured in light). **(D)** The protein levels of both arrestin and rhodopsin also increased in retinal neurocytes cultured in darkness. **(E)** Relative protein expression in retinal neurocytes was quantified by densitometry. The protein levels of rhodopsin and arrestin in primary retinal neurocytes cultured in darkness were significantly higher than those in the controls, which is consistent with the Crx level (**P < 0.001). All results were confirmed by three independent experiments. The error bars represent the SD of the mean (*n* ≥ 3). The asterisks represent statistically significant differences between the controls and experimental groups.

**FIGURE 4:**
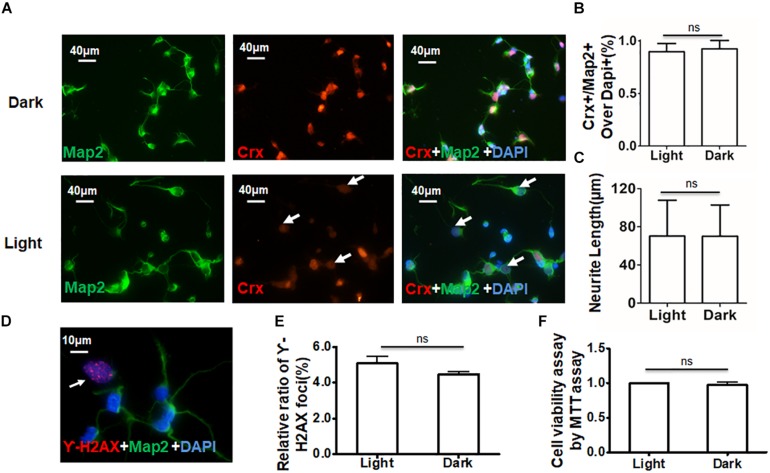
Daylight does not affect cell viability in primary retinal neurocytes. Suitable light stimulation did not affect the viability of retinal neurocytes. **(A)** Primary rat retinal neurocytes cultured in light or darkness were stained for Crx (red) and Map2 (green), and the nuclei were stained with DAPI (blue); the staining of Crx was weaker in the light stimulation group (white arrowheads). **(B)** The relative ratio of Crx- and Map2-positive cells over all cells (nuclei were stained with DAPI) showed no significant difference between the two groups (*p* > 0.1). **(C)** Neurite tracing in Crx- and Map2-positive neurons showed no significant difference in neurite length in the two groups (*p* > 0.1). **(D)** Primary rat retinal neurocytes cultured in light or darkness were stained for γ-H2AX (red) (white arrowheads) and Map2 (green), and the nuclei were stained with DAPI (blue). **(E)** The relative ratio of H2AX foci showed no significant difference in DNA damage between the two groups (*p* > 0.1). **(F)** Cell viability was determined by MTT assay and showed no significant difference between the two groups (*p* > 0.1). All results were confirmed by three independent experiments. The error bars represent the SD of the mean (*n* ≥ 3).

As a novel Otx-like paired-homeodomain protein, Crx may transactivate many photoreceptor cell-specific genes, such as rhodopsin, opsin, and arrestin (Yanagi et al., 2002; Hershey et al., 2012; Soto et al., 2012; Schueren et al., 2014). To further confirm that Crx is posttranscriptionally regulated by light stimulation in retinal neurocytes, we analyzed the protein levels of downstream genes rhodopsin and arrestin. As shown in Figures 3A,B, Crx was coexpressed with arrestin and rhodopsin in retinal neurocytes. Then, primary retinal neurocytes were cultured in light or darkness. Twenty-four hours after treatment, whole protein was extracted and analyzed by Western blot. The protein levels of rhodopsin and arrestin were also markedly increased (Figures 3C,D). The relative band intensities were quantified by densitometry and normalized to β-actin levels. Figure 4E shows that arrestin and rhodopsin levels were significantly increased in retinal neurocytes cultured in darkness compared with those cultured in light (Crx: light, 0.324 ± 0.096; dark: 0.894 ± 0.161; arrestin: light, 0.545 ± 0.339; dark, 1.107 ± 0.262; rhodopsin: light, 0.475 ± 0.215; dark, 0.878 ± 0.179; *P* < 0.05) (Figure 3E). The expression patterns of rhodopsin and arrestin were in accordance with Crx expression, which further suggests that the level of Crx protein is affected by light.

To ensure that the increased protein levels of Crx, rhodopsin, and arrestin were not caused by DNA damage or a change in cell viability due to light, three experiments were performed to characterize the retinal neurocytes *in vitro*. Double-staining for Crx and Map2 showed that the staining of Crx was weaker in the light stimulation group (white arrowheads) (Figure 4A). The ratio of neurons expressing Crx and Map2 and the neurite length were not significantly different between primary retinal neurocytes cultured in light and those cultured in the dark (Figures 4B,C). This suggested that light stimulation did not reduce the amount of Crx-positive neurons (Light, 89.67 ± 7.82; Dark, 92.56 ± 7.51%; *P* > 0.1) or inhibit the neurite outgrowth (Light, 70.22 ± 37.51 μm; Dark, 70.03 ± 32.84 μm; *P* > 0.1). Double-staining for γ-H2AX (white arrowheads), a specific DNA damage marker (Chen et al., 2019), and Map2 showed that light did not cause extra DNA damage compared with that caused by darkness (light, 5.073 ± 0.730%; dark, 4.453 ± 0.0460.271%; *P* > 0.1) (Figures 4D,E). Moreover, the MTT assay proved that light did not affect cell viability (light, 1; dark, 0.978 ± 0.063; *P* > 0.1) (Figure 4F). Therefore, the above results suggest that light stimulation affects the level of Crx protein in retinal neurocytes.

### Light Stimulation May Inhibit the Post-transcription of Endogenous Crx in Retinal Neurocytes

**FIGURE 5:**
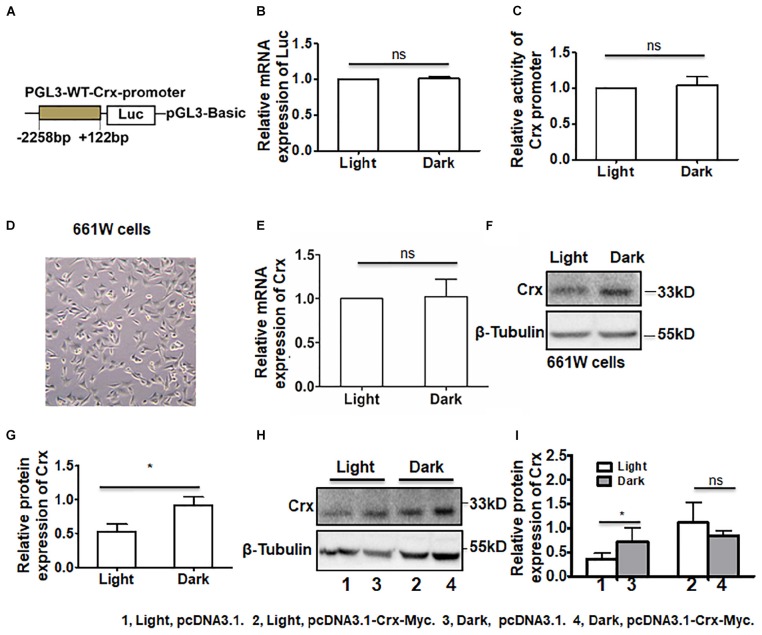
Posttranscriptional regulation by light stimulation is specific to the endogenous Crx gene. **(A)** The structure of the luciferase plasmid, which contained a rat Crx promoter sequence from −2258 to +122 bp. **(B)** Real-time RT-PCR indicated that the mRNA expression level of Luc in retinal neurocytes cultured in darkness was similar to that in retinal neurocytes cultured in light (*P* > 0.1). **(C)** The relative activity of the Crx promoter was quantified by a luciferase activity assay. Compared to darkness, light did not affect the luciferase activity of the Crx promoter (*P* > 0.1). **(D)** The morphology of 661W cells. **(E)** Crx mRNA expression in 661W cells was not affected by light (*p* > 0.1). **(F)** The levels of Crx protein expression in 661W cells were quantified by Western blot. **(G)** Crx protein expression in 661W cells cultured in darkness was significantly higher than in the controls (661W cells cultured in light) (*P < 0.05). **(H)** Endogenous and combined endogenous and exogenous Crx expression in 661W cells was analyzed by Western blot. **(I)** Light affected endogenous Crx protein expression (*P < 0.05) but did not decrease combined endogenous and exogenous Crx protein expression (*P* > 0.1). All results were confirmed by three independent experiments. The error bars represent the SD of the mean (*n* ≥ 3). The asterisks represent statistically significant differences between the controls and experimental groups.

To further demonstrate the results above, we analyzed the activity of the Crx promoter in retinal neurocytes cultured in light or darkness. We generated a construct containing a mouse Crx promoter sequence between positions −2258 and + 122 bp and a luciferase reporter gene (Figure 5A). Primary retinal neurocytes cultured in light or darkness were transfected with the construct. Twenty-four hours after transfection, we measured luciferase mRNA in the primary retinal neurocytes by quantitative RT-PCR. As shown in Figure 5B, the levels of luciferase mRNA were not different in cells cultured in light and those cultured in the dark (light, 1; dark, 1.01 ± 0.046; *P* > 0.1). Moreover, a change in luciferase activity in cells cultured in light and those cultured in the dark was also not observed (Light, 1; Dark, 1.044 ± 0.227; *P* > 0.1) (Figure 5C). The level of Luc protein was consistent with that of Luc mRNA. Therefore, these results further indicate that light does not affect Crx promoter activity, which supports the notion that light can inhibit the post-transcription of the Crx gene.

In addition, we asked whether has exogenous Crx had the same expression pattern and whether light affected Crx stability in cells. Due to low transfection efficiency in primary retinal neurocytes, a construct containing the CMV promoter and mouse Crx cDNA-Myc was transfected into 661W cells, a photoreceptor-derived cell line. First, we detected the mRNA and protein expression levels of endogenous Crx in 661W cells. As shown in Figure 5E, light treatment did not change the level of Crx mRNA (light, 1; dark, 1.022 ± 0.393; *P* > 0.1). Moreover, the level of Crx protein was consistent with that in 661W cells (Figure 5F). We calculated the density of the Western blot bands and found that the protein level of Crx was significantly higher in 661W cells cultured in darkness than in 661W cells cultured in light (light, 0.524 ± 0.227; dark, 0.917 ± 0.242; *P* < 0.05, Figure 5G). This inconsistency between Crx mRNA and protein levels in 661W cells is consistent with that in primary retinal neurocytes. Additionally, the activity of Crx promoter in 661w cells did not show significant difference between the cells cultured in light and dark (Supplementary Figure S2). Twenty-four hours after the transfection of the plasmids expressing Crx and vehicle, whole protein and total RNA were extracted from cells cultured in light or dark. Western blotting was performed to analyze endogenous and exogenous Crx expression in the cells (Figure 5H). The relative expression of Crx was quantified by densitometry. As shown in Figure 5I, the protein level of endogenous Crx was also increased in cells cultured in the dark (light, pcDNA3.1, 0.362 ± 0.114; dark, pcDNA3.1, 0.710 ± 0.288, *P* < 0.05). However, the combined level of endogenous and exogenous Crx protein in 661W cells cultured in the dark was consistent with that in cells cultured in the light after being transfected with the plasmid-expressing exogenous Crx (light, pcDNA3.1-Crx, 1.112 ± 0.414; dark, pcDNA3.1-Crx, 0.830 ± 0.123, *P* > 0.1). Therefore, these results suggest that the posttranscriptional regulation of Crx by light stimulation is specific to the expression of the endogenous Crx gene. Moreover, light does not impact the stability of the Crx protein in cells.

### Crx Is Translationally Regulated in the Developing Retina

**FIGURE 6:**
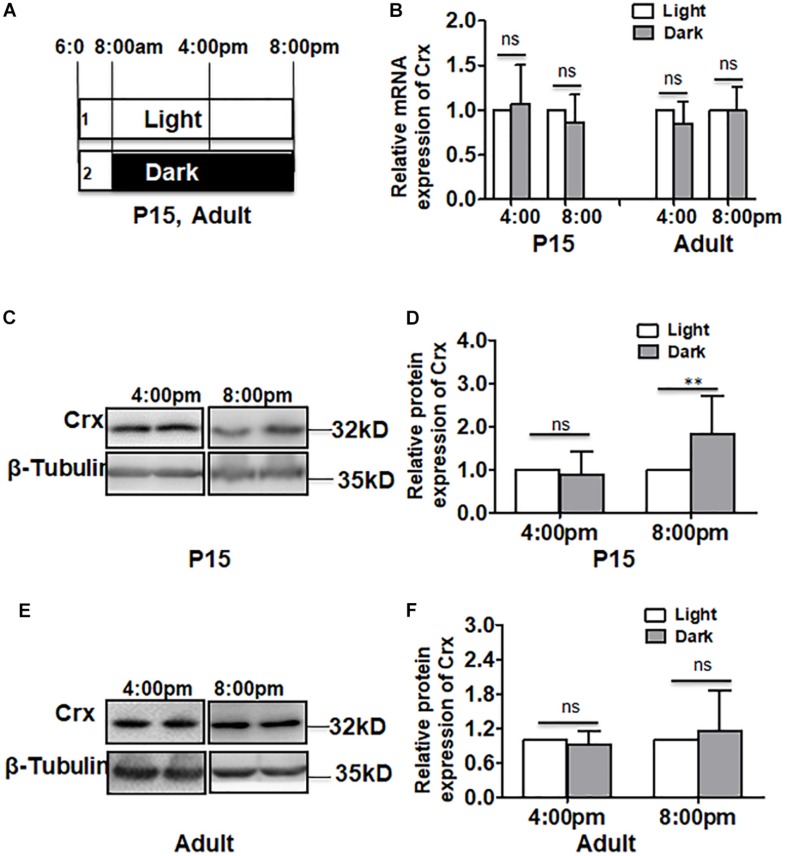
Crx is translationally regulated in the retina *in vivo*. **(A)** The rat treatment strategy for analyzing the mRNA and protein levels of Crx *in vivo*. Adult and P15 rats were exposed to ordinary diurnal light from 8:00 am, and their right eyes were covered with eyepatches. The contralateral eyes were used as controls. mRNA and protein were collected from the retinas at 4:00 pm and 8:00 pm. **(B)** RT-PCR showed that the mRNA level of Crx in the retinas of the covered eyes and the contralateral eyes was not affected by light at 4:00 pm and 8:00 pm in either adult or P15 rats (*P* > 0.1). **(C)** Western blotting showed that Crx protein levels increased in the retinal tissues of the covered eyes of P15 rats and in those of the contralateral eyes. **(D)** The relative expression of Crx in retinal neurocytes was quantified by densitometry. The Crx protein level did not change in the retinal tissues of the covered eyes of P15 rats compared to that in the contralateral eye at 4:00 pm (*P* > 0.5) but increased at 8:00 pm (**P < 0.001). **(E)** Crx protein levels in the retinal tissues of adult rats were assayed by Western blot. **(F)** The Crx protein level in the retinal tissues of the covered eyes was the same as that in the contralateral eyes (exposed to light). In adult rats, Crx protein expression was not affected by daylight at either 4:00 pm or 8:00 pm (*P* > 0.5). All results were confirmed by three independent experiments. The error bars represent the SD of the mean (*n* ≥ 3). The asterisks represent statistically significant differences between the controls and experimental groups.

We next asked whether Crx in retinal neurocytes responds to light stimulation *in vivo* as it does *in vitro*. To answer this question, rats were reared in the conditions shown in Figure 6A. Adult and postnatal day 15 (P15) rats were reared in the light with one eye covered by an eyepatch. This strategy of treatment prevents the control of the circadian clock (Schueren et al., 2014). We measured the mRNA expression and protein levels of Crx in retinal tissues in postnatal day 15 (P15) and adult rats at 4:00 pm and 8:00 pm by quantitative RT-PCR and Western blot. As shown in Figure 6B, the level of Crx mRNA at different time points remained the same in the retinas of P15 rats reared in the dark or light (4:00 pm: light, 1; dark, 1.067 ± 0.439; 8:00 pm: light, 1; dark, 0.858 ± 0.316, *P* > 0.5, Figure 6B). These results were also observed in adult rats (4:00 pm: light, 1; dark, 0.851 ± 0.251. 8:00 pm: light, 1; dark, 1.001 ± 0.258, *P* > 0.1, Figure 6B). Consistent with the *in vitro* results, the Crx protein level at 8:00 pm was significantly increased in the retinas of P15 rats reared in darkness (8:00 pm: light, 1; dark, 1.668 ± 0.46, *P* < 0.001), although it was the same as that observed at 4:00 pm (4:00 pm: light, 1; dark, 0.940 ± 0.49, *P* > 0.5, Figures 6C,D). However, an alteration in Crx protein expression was not observed in the retinas of adult rats reared in darkness or light (4:00 pm: light, 1; dark, 0.922 ± 0.238. 8:00 pm: light, 1; dark, 1.145 ± 0.718, *P* > 0.5, Figures 6E,F).

**FIGURE 7:**
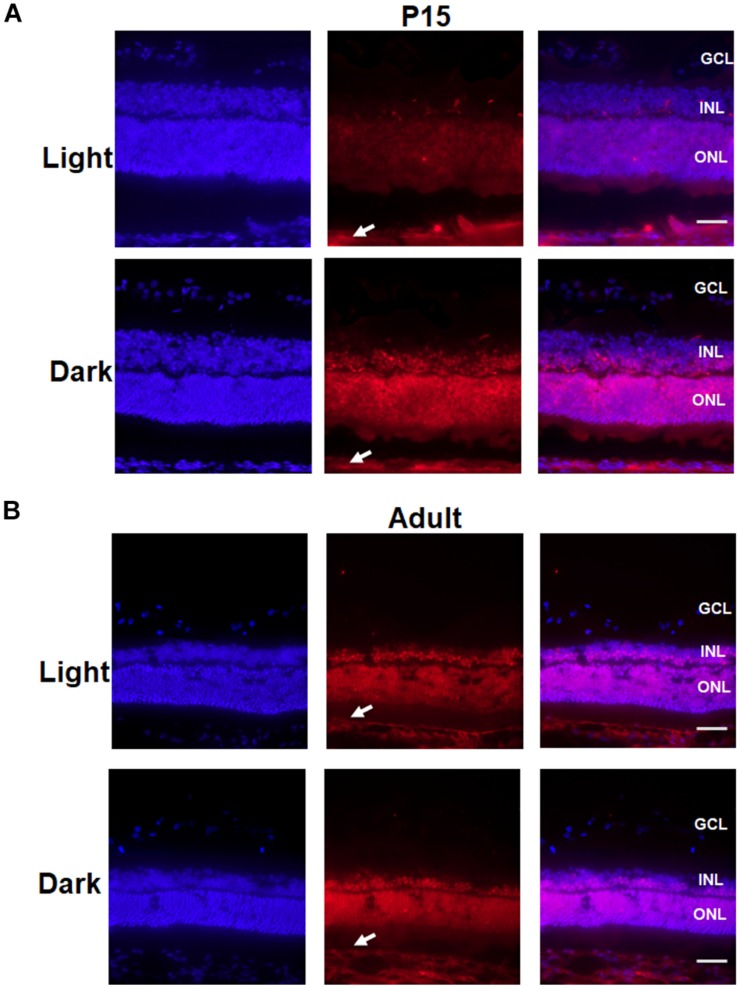
Change in the localization of Crx in the P15 and adult rat retina. P15 and adult rat retinas were stained for Crx (red) and DAPI (blue). **(A)** Crx expression in the P15 rat retinal inner nuclear lays (INL) and outer nuclear lays (ONL) of the eyes covered with eyepatches was notably stronger than that in the contralateral eyes, while the non-specific staining in sclera was the same in covered and un-covered retina (white arrowheads). **(B)** Crx expression in the adult retina in the covered and the un-covered group did not show significant difference, while non-specific staining in sclera was the same in two groups (white arrowheads). Scale bars: 100 μm.

Moreover, an immunofluorescence assay was performed to determine the location in which Crx expression was altered in the retinas of P15 rats. As shown in Figure 7, Crx was mainly expressed in both the outer nuclear layer (ONL) and inner nuclear layer (INL) of the retina. However, there was notably stronger Crx expression in the INL and ONL of the eyes that were covered with eyepatches compared with that in the contralateral eye, as shown by the non-specific positive staining in sclera (white arrowheads) (Figure 7A). This result was not observed in the adult retina (Figure 7B). Therefore, a posttranscriptional regulatory mechanism may be involved in producing inconsistent mRNA and protein levels of Crx in the developing retina.

## Discussion

A growing body of evidence indicates that daylight exposure is an important factor for optical development (Rao et al., 2013). The retina is a key component of the visual system. Thus, the elucidation of the mechanism by which Crx is expressed in the retina is meaningful. Currently, the expression pattern of Crx in the pineal gland, in which Crx transcript levels exhibit a circadian rhythm with a peak in the middle of the night (Li et al., 1999), is well understood. In this study, we focused on how light stimulation affects Crx expression in retinal neurocytes. Therefore, an experiment was designed to avoid control of the circadian clock, as shown by the data in Figure 1. At 24 h after L/D or D/D treatment, the level of Crx mRNA and protein was similar (Figures 1C,E). However, if the cells were cultured in light or darkness, respectively, Crx protein was significantly increased in cells cultured in darkness (Figure 2D). Light exposure did not affect Crx promoter activity (Figure 5). Thus, these results indicate that light stimulation could suppress Crx protein expression without changing the mRNA level. Moreover, comparing this with the results shown in Figures 1E, 2D, we can see that the expression of Crx protein returned to the original level after 12 h cultured in darkness, so we could not observe the effect of Light/Dark circadian rhythm on Crx expression when the whole RNA and protein were extracted under darkness (Figure 1). In addition, the expression pattern of downstream genes rhodopsin and arrestin is also downregulated in retinal neurocytes cultured in light (Figure 3). Therefore, our data suggest that Crx is posttranscriptionally regulated by light stimulation in retinal neurocytes. Previous studies that showed that Crx mRNA is stable and that downstream NAT mRNA in the retina is altered in the daytime versus the nighttime (Rohde et al., 2014) partially support our discovery.

Moreover, our data suggest that light stimulation only affects endogenous Crx expression in retinal neurocytes. We transfected an expression plasmid into 661W cells. Our data show that endogenous Crx exhibited inconsistent mRNA and protein levels (Figures 5E–G), whereas the combined exogenous and endogenous Crx level was not affected by light stimulation (Figures 5H,I). These results also indicate that light stimulation does not impair Crx protein stability. The decrease of Crx protein induced by light stimulation seems to be associated with a reduction in protein synthesis rather than a higher degradation rate. Therefore, the results above strongly indicate that the posttranscriptional mechanism is specific to the Crx gene in retinal neurocytes.

Furthermore, our results also suggest that the posttranscriptional regulation mechanism may be involved in Crx expression in the developing retina. As shown in Figure 6, the inconsistent expression of Crx mRNA and protein stimulated by light occurred in the retinas of postnatal day 15 rats but not in the retinas of adult rats. Contrary to the *in vitro* results, the mRNA and protein levels of Crx were the same in the adult retinas that were covered with eyepatches and the contralateral retinas (Figures 6B,F). Only the results obtained from the retinas of P15 rats are consistent with the *in vitro* results, in which the level of Crx mRNA in the retina of both eyes was equal, and the Crx protein level was significantly higher in the retina of the eye exposed to the dark compared to the contralateral eye (Figures 6B,D). Moreover, primary retinal neurocytes and 661W cells are premature cells (Meola et al., 2012; Wang et al., 2018). Rats usually open their eyes on postnatal day 15, which indicates that the retina is developing at that time. Therefore, both cells have the same pattern of Crx expression as that of P15 rats (Figures 2, 5E,F). Thus, we conclude that the posttranscriptional regulatory mechanism of Crx expression is involved in the development of premature retinal neurocytes. This notion is also partially supported by a previous study. A comprehensive international study demonstrated that exposure to bright outdoor light plays a more important role than physical activity in protecting the vision of the younger generation (He et al., 2015).

In addition, our data provide three ideas for future studies. First, as described above, Crx is downregulated in premature rat retinas by daylight stimulation-induced posttranscriptional regulation. However, posttranscriptional regulation is a complicated process. Common posttranscriptional regulatory elements include upstream open reading frames, codon optimality, start codon context, etc. (Malys and McCarthy, 2011; Hershey et al., 2012; Schueren et al., 2014). More investigations of Crx-specific posttranscriptional regulation are required. Secondly, Crx plays a key role in a regulatory network in the retina. It has been reported that Crx regulates more than 700 genes in photoreceptor-specific cells (Mansergh et al., 2015). For instance, Crx regulates rod photoreceptor differentiation by collaborating with Nrl and Nr2e3 (Chow et al., 1998; Peng et al., 2005; Roberts et al., 2006) and works together with Rorβ2 and Trβ2 to modulate cone photoreceptor differentiation and development (Organisciak et al., 1998; Furukawa et al., 1999; Arcot Sadagopan et al., 2015). However, Crx translation is regulated by daylight stimulation. Therefore, a daily light-dark cycle should be considered in the design of future *in vitro* and *in vivo* experiments involving the retina to ensure accurate results *in vivo*. Lastly, previous studies have demonstrated that changes in rhodopsin and arrestin expression are involved in the mechanism of myopia development (Hu et al., 2008). Both rhodopsin and arrestin are upregulated in the retinas of rats reared in the dark (Dryja et al., 1990; Paluru et al., 2005; Craft and Deming, 2014). On the other hand, decreased outdoor activity induces myopia and promotes myopia progression in juvenile-onset myopes (Jones-Jordan et al., 2012; Wu et al., 2013). Therefore, we speculate that high levels of Crx protein in retinal neurocytes cultured in darkness may be involved in a molecular mechanism of myopia formation. More investigations in this direction are also required.

## Conclusion

In conclusion, the evidence presented here reveals a novel mechanism by which Crx is posttranscriptionally regulated by light stimulation in postnatal rat retina. This study not only sheds light on the underlying mechanism of Crx expression in the retina but also suggests that light conditions should be taken into consideration when designing experiments in retinal developmental study.

## Data Availability Statement

The raw data supporting the conclusions of this article will be made available by the authors, without undue reservation, to any qualified researcher.

## Ethics Statement

The animals were treated in accordance with the ARVO Statement for the Use of Animals in Ophthalmic and Vision Research. The protocol was approved by the Animal Care and Use Committee of Sun Yat-sen University.

## Author Contributions

JZhu and KY conceived the study. YW and JQ design the experiments. YW, JQ, XC, SC, PZ, JjZ, and MY performed majority of the experiments. HC, SL, JZha, and JG contributed to data analysis. JZhu and KY supervised all experiments and wrote the manuscript. All authors read and approved the manuscript.

## Conflict of Interest

The authors declare that the research was conducted in the absence of any commercial or financial relationships that could be construed as a potential conflict of interest.
