# Supplementary figures and images for: Crx Is Posttranscriptionally Regulated by Light Stimulation in Postnatal Rat Retina
Source: Front Cell Dev Biol. 2020 Apr 7;8:174. doi: 10.3389/fcell.2020.00174 (PMC7154164; doi:10.3389/fcell.2020.00174)

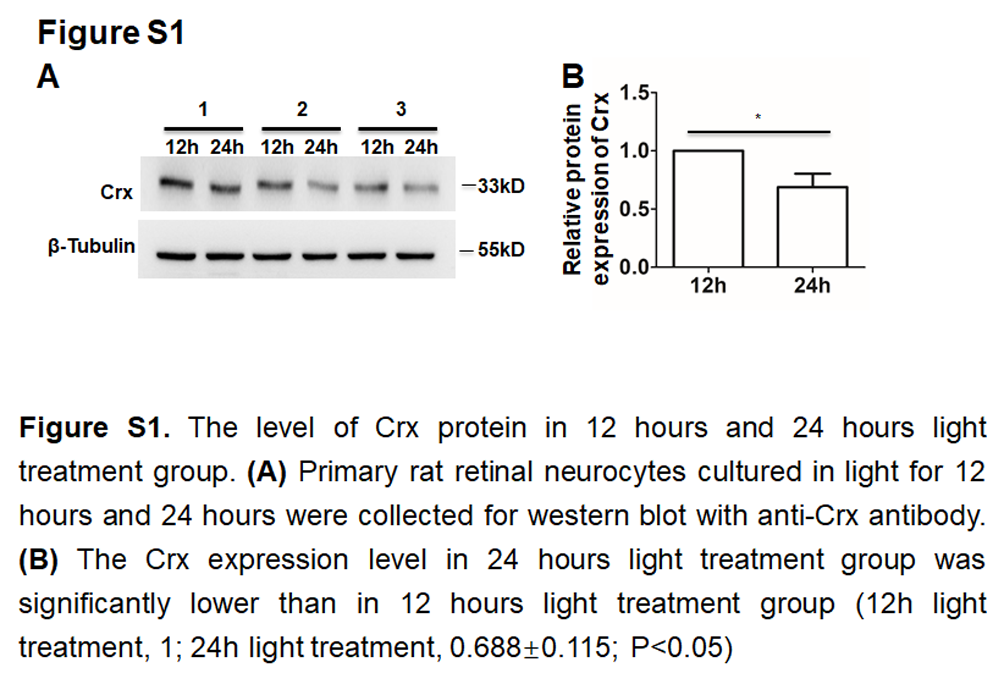

Supplement: Supplementary file 1 [file Image_1.TIF]

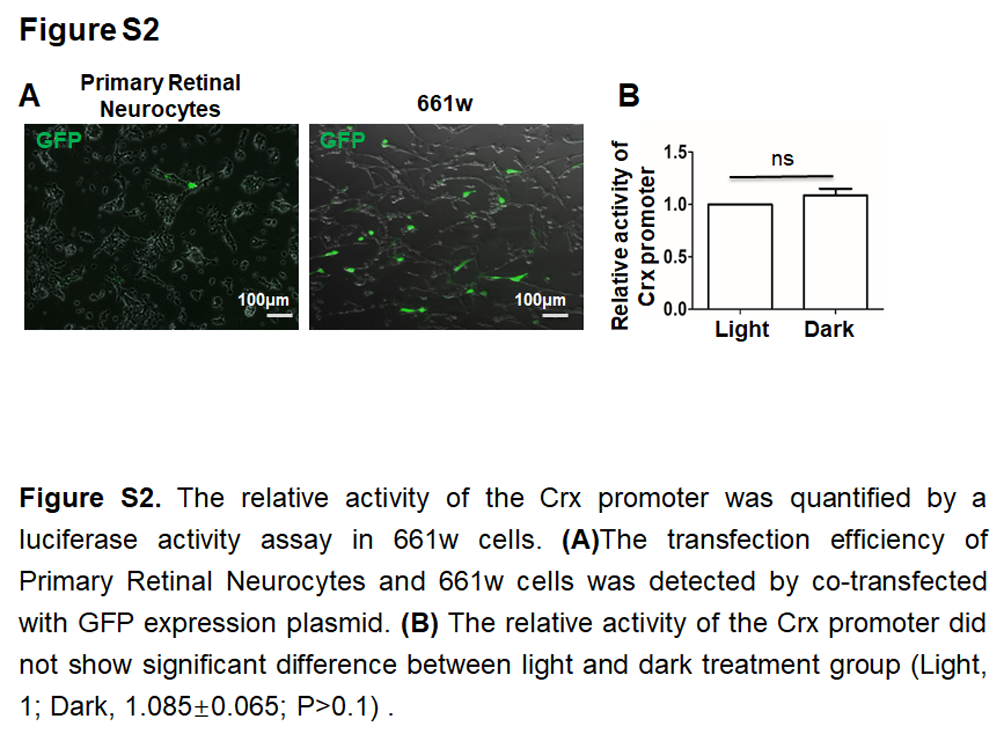

Supplement: Supplementary file 2 [file Image_2.TIF]
